# Integrating cerebrovascular morphology and radiomics features for predicting stroke prognosis: a retrospective study

**DOI:** 10.7717/peerj.20588

**Published:** 2026-01-09

**Authors:** Suying Pu, Shunjun Li, Jing Shao, Jixian Lin, Huanyin Li, Jinjiang Shen, Hui Zheng

**Affiliations:** 1 Shanghai Xuhui District Dahua Hospital, Shanghai, China; 2 Minhang Hospital, Fudan University, Shanghai, China

**Keywords:** Stroke, Vascular structure, Radiomics, Machine learning, 90-day mRS prediction prediction

## Abstract

Accurately predicting 90-day Modified Rankin Scale (mRS) scores for acute ischemic stroke (AIS) patients is crucial for guiding treatment strategies. However, many existing mRS prediction methods rely on clinicians to manually evaluate relevant features, and the accuracy of feature quantification and model reproducibility still need to be further improved. This study proposes a machine learning framework that combines multimodal imaging features in order to predict 90-day mRS outcomes. A retrospective analysis was conducted on 86 AIS cases. Morphological features of the intracranial arterial and venous system were extracted from computed tomography angiography (CTA) images. Additionally, radiomics features were obtained from the ischemic lesion on diffusion-weighted imaging (DWI). Recognizing the significance of the peri-infarct penumbra in stroke prognosis, radiomics features were also extracted from the annular region surrounding the ischemic lesion. Redundant features were eliminated using a sparse representation method, and a sparse representation-based classifier was developed to predict mRS outcomes. Model performance was validated using cross-validation and independent test. A total of 1,066 features, including 40 vascular morphological features and 1,026 radiomics features, were extracted. Both feature types demonstrated statistical significance (*P* < 0.05). Ultimately, 26 features were selected to construct the classification model. The proposed model achieved robust performance on the independent test set, with a classification accuracy of 0.828, an area under the curve (AUC) of 0.942, sensitivity of 0.789, specificity of 0.900, positive predictive value of 0.937, and negative predictive value of 0.692. By integrating vascular morphological features with radiomics features from the ischemic lesion and peri-ischemic lesion regions in DWI, the proposed machine learning model provides accurate predictions of 90-day clinical outcomes for AIS, offering valuable insights for personalized stroke management.

## Introduction

Stroke is one of the leading causes of morbidity and mortality worldwide, resulting in significant long-term consequences for both patients and healthcare systems (Mistry et al., 2021). The Modified Rankin Scale (mRS) is widely used to assess the functional outcome of stroke patients, and predicting the 90-day mRS score is critical for guiding clinical decision-making and rehabilitation strategies (Xu et al., 2021). Accurate prediction of post-stroke functional outcomes can help identify patients at high risk of poor recovery, allowing for targeted interventions and resource allocation (Huo et al., 2023). Moreover, this prediction has the potential to enhance prognostic models, aid in the evaluation of new therapies, and improve patient counseling and planning. Recent advances in neuroimaging and machine learning techniques have shown promising results in improving the accuracy of 90-day mRS outcome predictions. Imaging biomarkers, such as lesion volume, location, and the presence of collateral circulation, have been identified as critical predictors of functional recovery. However, challenges remain in optimizing these models for clinical use, and a better understanding of the underlying factors contributing to functional outcomes is essential for improving stroke rehabilitation and personalized treatment strategies (Xu et al., 2021; Mistry et al., 2021; Huo et al., 2023).

Collateral circulation, an alternative blood flow pathway that sustains and protects brain tissue post-stroke, plays a pivotal role in determining stroke prognosis (Wang et al., 2013; Chen et al., 2023). The structural morphology of cerebral blood vessels plays a crucial role in collateral circulation, and numerous studies have investigated the predictive value of vascular morphology in stroke outcomes. For example, Lei et al. (2024) explored the association between enlarged perivascular spaces (PVS) and outcomes of intravenous thrombolysis in acute ischemic stroke (AIS) patients, while Zhang et al. (2017) examined the relationship between the total magnetic resonance imaging (MRI) burden of cerebral small vessel disease (SVD) and post-stroke depression in patients with acute lacunar stroke. Van Der Hoeven et al. (2016) demonstrated that superior collateral circulation was correlated with better 90-day mRS outcomes in patients undergoing endovascular therapy, underscoring its importance in prognosis. Despite these achievements, limitations such as manual assessment bias and limited quantitative analysis continue to restrict clinical translation. Many existing methods rely on manual vascular assessments performed by radiologists, which introduces subjectivity and variability due to differences in expertise and experience, thereby reducing reproducibility. Furthermore, these studies often lack a comprehensive and quantitative vascular assessment, which restricts their clinical applicability and predictive performance (Weng et al., 2023).

Simultaneously, the ischemic lesion on diffusion-weighted imaging (DWI) has also emerged as a key focus for stroke prognosis research. Li et al. (2025) extracted 851 features of the ischemic lesion to predict 90-day mRS outcomes, while Wei et al. (2024) analyzed imaging features from DWI and apparent diffusion coefficient (ADC) maps to identify AIS patients at high risk of poor recovery. Wang et al. (2022) utilized DWI-based radiomics to predict 1-year ischemic stroke recurrence. However, these methods often focus exclusively on the ischemic lesion and neglect the surrounding penumbra, which is a critical target for therapeutic intervention (Vagal et al., 2018). Although both the ischemic lesion and penumbra are closely linked to stroke prognosis (Tang et al., 2020), their characterization fundamentally depends on the global morphology of the intracranial arterial and venous system. A combined assessment that integrates these dimensions may provide a more comprehensive understanding of stroke pathology and improve outcome predictions.

In this study, we propose a novel machine learning framework that integrates whole-brain vascular morphological features with radiomics features of both the ischemic lesion and peri-infarct region to predict 90-day mRS outcomes. Specifically, our objectives are: (1) to systematically quantify cerebral vascular morphology, (2) to extract radiomics features from the ischemic lesion and peri-ischemic lesion regions, and (3) to construct and validate a sparse representation-based multimodal predictive model.

## Materials & Methods

### Materials

**Figure 1:**
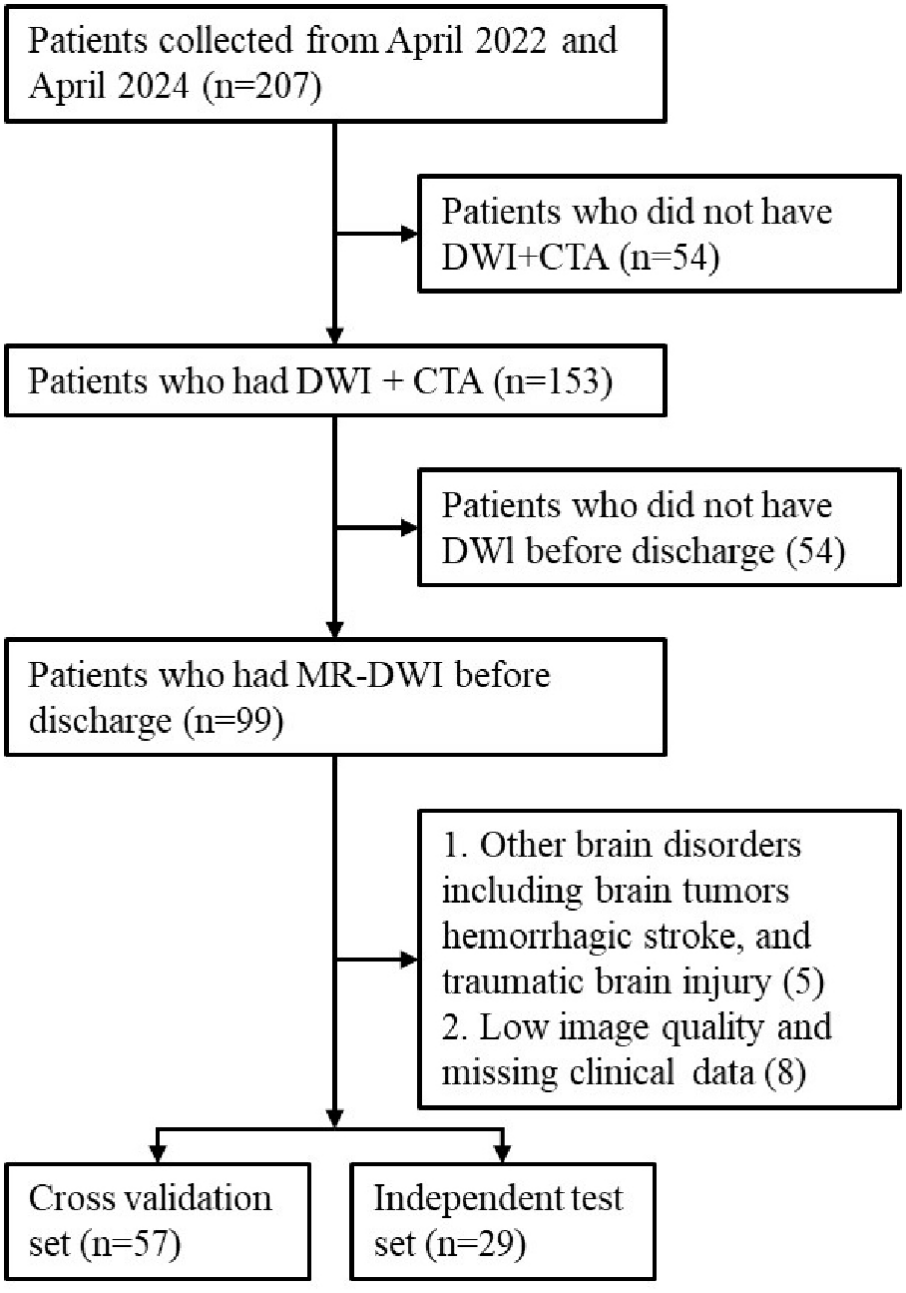
The patient selection flowchart.

A total of 207 patients with acute cerebral infarction hospitalized in the Department of Neurology of Xuhui District Dahua Hospital and Minhang Hospital, Fudan University between April 2022 and April 2024 were included in this study. All these cases met the diagnostic criteria for acute cerebral infarction, were diagnosed using imaging such as cranial computed tomography (CT) or cranial MRI and neurological physical examination, and had NIHSS scores ranging from 0 to 25. Inclusion criteria were: (1) Case diagnosed as AIS; (2) Pre-treatment computed tomography angiography (CTA) imaging; (3) DWI imaging performed within 24 h of admission; (4) Complete clinical data. Exclusion criteria were: hemorrhagic stroke, brain tumors, poor image quality, or missing data. Thrombolysis or thrombectomy status (TICI score) was not used as an inclusion criterion, as our aim was to construct a model applicable across the entire AIS population. Figure 1 provides the patient selection flowchart. Eighty-six cases were ultimately included in our experiment. These data were randomly divided into a cross-validation set and an independent testing set at a ratio of 2:1. Based on existing studies (Seker et al., 2020) on 90-day mRS predictions, we defined a 90-day mRS score of less than 3 as a good prognosis and scores of 3 or higher as a bad prognosis, and then built two classification models to predict these outcomes.

In this retrospective study, all patients underwent non-contrast CT and CTA at admission for emergency diagnosis and treatment decision-making in accordance with standard AIS protocols. MRI examinations including DWI and ADC sequences were performed within two days after hospitalization for post-treatment evaluation and radiomic analysis (Li et al., 2025). No CT perfusion (CTP) imaging was used in this study.

The CTA images were acquired on Siemens Sensation 64 with the following parameters: tube voltage of 80 kV, tube current of 50 mAS, and slice thickness 10 mm. The voxel resolution and size of the CTA images are 0.486 mm * 0.486 mm * 1 mm and 512 * 512 * 319, respectively. The DWI images were acquired on a Magnetom Trio 3T GE scanner with the following parameters: (TR/TE=2800/75.4 msec; FA=90°; Slice thickness/gap=5/1.5 mm; FOV=230 mm×220 mm). The voxel resolution and size of the DWI images are 0.9375 mm * 0.9375 mm * 6 mm and 256 * 256 * 16, respectively.

### Methods

**Figure 2:**
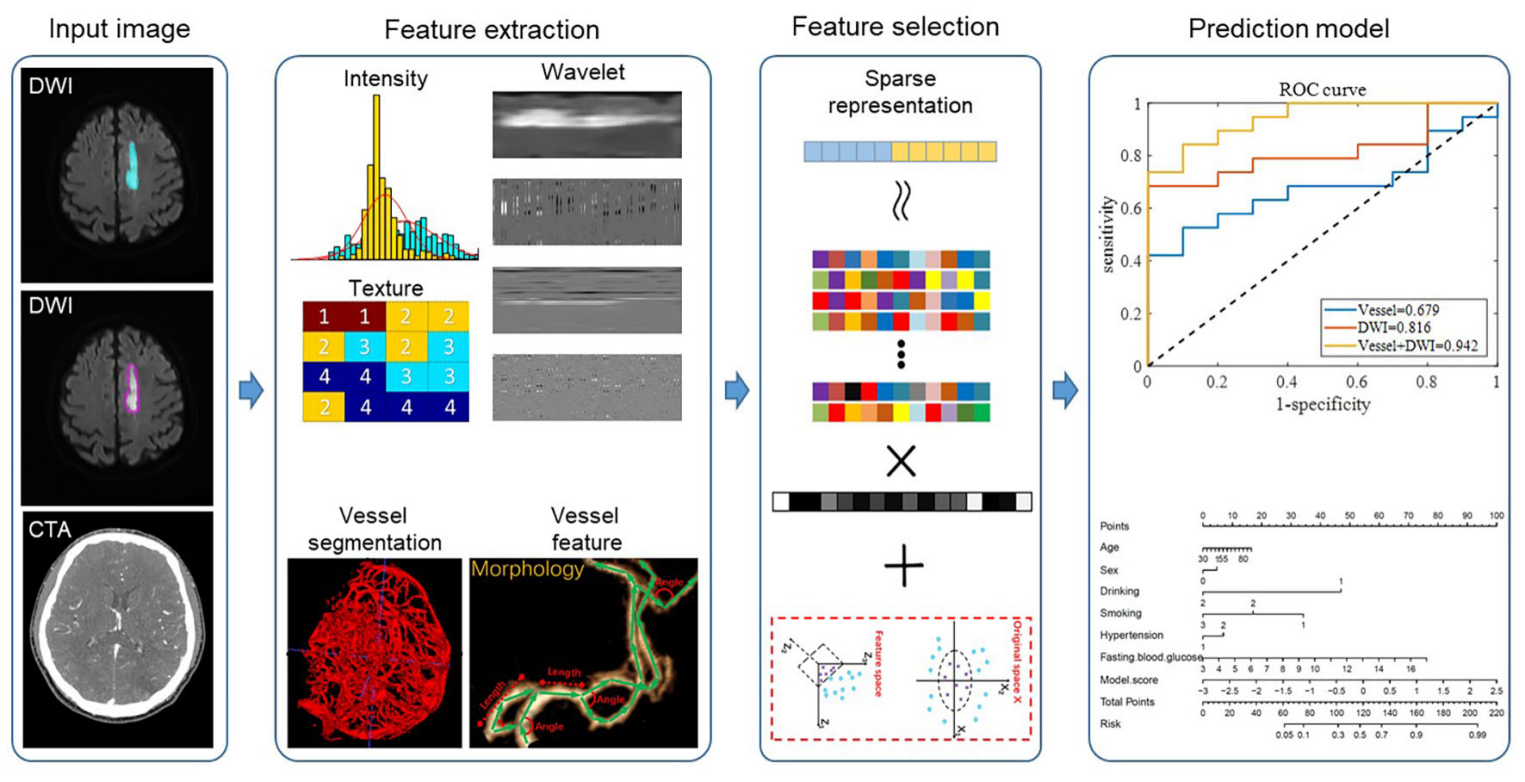
The workflow of the proposed framework.

The overall framework of our proposed method is shown in Fig. 2. For the input DWI images, we extracted radiomic features from both the ischemic lesion and peri-ischemic lesion regions. For the input CTA images, we first automatically segmented the whole brain vessels and then extracted the morphological features of the vessels. After the radiomic features and vascular features were fused, the features were filtered using the sparse representation method. Sparse representation is a commonly used feature selection method. It uses the features of samples in the training set to linearly represent the class labels and selects the optimal feature combination to represent the sample labels by sparsifying the representation coefficients. Sparse representation can select a set of features that are highly relevant to the sample class and have low inter-feature correlation, thereby improving model classification performance and reducing model overfitting. (For the specific mathematical expression model of sparse representation feature selection, please refer to Wu et al. (2019b)). Finally, the filtered features were sent to the classifier to predict the quality of the 90-day mRS scores. In each of the following subsections, we described each step in detail. This retrospective study was approved by the Institutional Ethics Committee of Xuhui District Dahua Hospital (Approval No. 20241008) and Minhang Hospital, Fudan University Approval No. 2021-Batch-008-01K. Written informed consent was obtained from all subjects (patients) in this study.

#### Feature extraction

**Figure 3:**
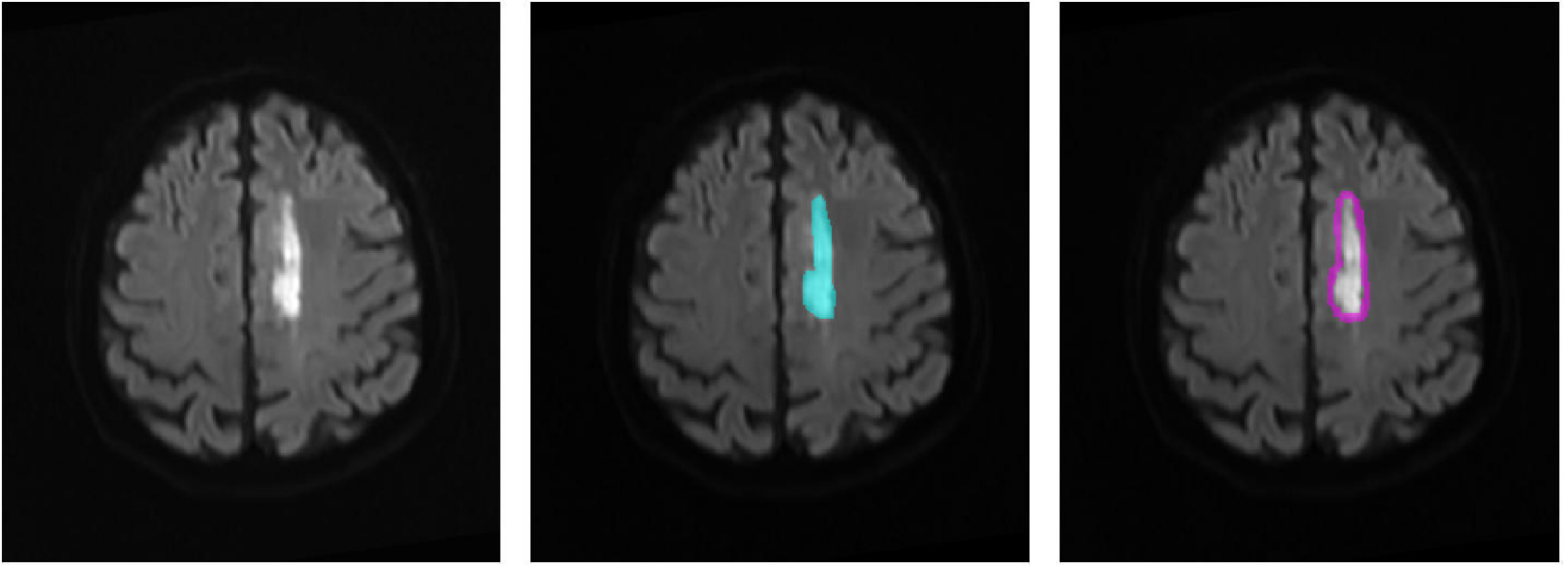
Region of interest on the DWI image. (1) The original DWI image (left), (2) the annotated ischemic lesion (middle), and (3) the delineated peri-ischemic region (right) used for radiomics analysis.

We defined the DWI lesion as the ischemic lesion. Because CT perfusion data were not available for all patients, the peri-ischemic penumbra was approximated by outwardly expanding the ischemic lesion mask on DWI by 10 pixels, following previous studies. This approximated penumbra was not used for direct clinical diagnosis, but only as a region of interest for subsequent radiomics analysis. Radiomics transforms imaging data into high-throughput features, enabling the discovery of imaging biomarkers associated with stroke prognosis. Figure 3 illustrates the original DWI image, manually delineated ischemic lesion, and calculated peri-ischemic region. The ischemic lesion region was annotated by two senior radiologists: one performed the annotation and the other verified its accuracy. We extracted three types of radiomics features from the ischemic lesion region and peri-ischemic region. Intensity features: 18 features quantifying the statistical distribution of voxel intensities within the lesion. Texture features: 39 features evaluating the spatial arrangement of the lesion, categorized into four subgroups (gray-level co-occurrence matrix, gray-level run-length matrix, gray-level size zone matrix, and neighborhood gray-tone difference matrix). Wavelet features: each feature was then decomposed into eight frequency sub-bands, resulting in 456 (*i.e.,* (18+39)∗8) wavelet features. In total, 513 (*i.e.,* 18+39+456) features were extracted, the detailed calculations of which refer to Vallières et al. (2015) and Wu et al. (2019a).

To analyze vascular morphology, a U-Net (Lv et al., 2023) model was employed to automatically segment cerebral vessels from CTA images. The 3D vessel centerline was then extracted to quantify vascular structural information. As shown in the second row of the feature extraction part of Fig. 2, based on the centerline, 10 vascular features were calculated: vessel volume, number of vessel branches, total vessel length, average vessel length, average distance factor (DF) (Ronneberger, Fischer & Brox, 2015), average sum of angles metric (SOAM) (Fu et al., 2020), number of edge nodes, number of network nodes, clustering coefficient, and structure entropy. To enhance detail, 2D vascular features were also extracted. First, the 3D vessels were projected onto the 2D axial plane. Blood vessel enhancement, 2D threshold segmentation, and centerline extraction were then performed to calculate 10 additional features. Stroke-induced asymmetry in the intracranial arterial and venous system was further analyzed by computing morphological differences between the affected and healthy hemispheres (affected side minus healthy side). In total, 40 morphological features of cerebral vascular structures were extracted, including both 3D and 2D features.

All vascular feature extraction steps were fully automated. No manual correction was required. Vascular features were analyzed independently from DWI radiomics, and integration was performed at the feature level rather than through spatial registration.

#### Feature selection and classification model

There was some redundant information in the 1,066 extracted features that could potentially increase computational complexity and risk of overfitting. To address this, a sparse representation-based feature selection method was applied. This approach retains features with the highest discriminative ability by sparsely representing each test sample with training features from different classes. Ultimately, 26 features were selected, as they achieved the best classification performance in cross-validation while maintaining model simplicity and interpretability. A classification model leveraging sparse representation was then built. During classification, test sample features were sparsely represented using the training set features from different classes, and residuals were calculated for each class. The test sample was assigned to the class with the minimum residuals (Wu et al., 2018). The dataset of 86 cases was randomly split into a 10-fold cross-validation set and an independent testing set at a 2:1 ratio.

#### Statistical analyses

Prior to model development, independent sample t-tests were performed to evaluate differences in extracted imaging features between the good- and poor-prognosis groups (*P* < 0.05 was considered statistically significant). Model performance was assessed using accuracy (ACC), sensitivity (SEN), specificity (SPE), positive predictive value (PPV), negative predictive value (NPV), and the area under the receiver operating characteristic curve (AUC). Differences in AUC between models were compared using the DeLong test.

## Results

The clinical statistical characteristics of the 86 final enrolled patients are shown in Table 1. There were 39 cases in the poor prognosis group (MRS>2), and 47 cases in the good prognosis group (MRS<3). Overall, the average age and risk factors of the poor prognosis group were higher than those of the good prognosis group. Of the enrolled cases, 82% of the patients who underwent thrombectomy achieved a Thrombolysis in Cerebral Infarction (TICI) score ≥ 2b, reflecting successful reperfusion in the majority of EVT cases.

**Table 1:** The statistical information of the 86 cases.

Variables	ALL	MRS > 2 (39)	MRS < 3 (47)
Age	64.16 ± 11.75	66.15 ± 10.25	62.51 ± 12.49
Sex, male (%)	64 (74.42)	27 (69.23)	37 (78.72)
Location of occlusion (%)			
Internal carotid artery	17 (19.77)	7 (17.95)	10 (21.28)
Middle cerebral artery	69 (80.23)	32 (82.05)	37 (78.72)
Risk factors (%)			
Hypertension	36 (41.86)	26 (66.67)	10 (21.28)
Diabetes	21 (24.42)	12 (30.77)	9 (19.15)
Smoke	54 (62.79)	28 (71.79)	26 (55.32)
Drinking	11 (12.79)	6 (15.38)	5 (10.64)

**Figure 4:**
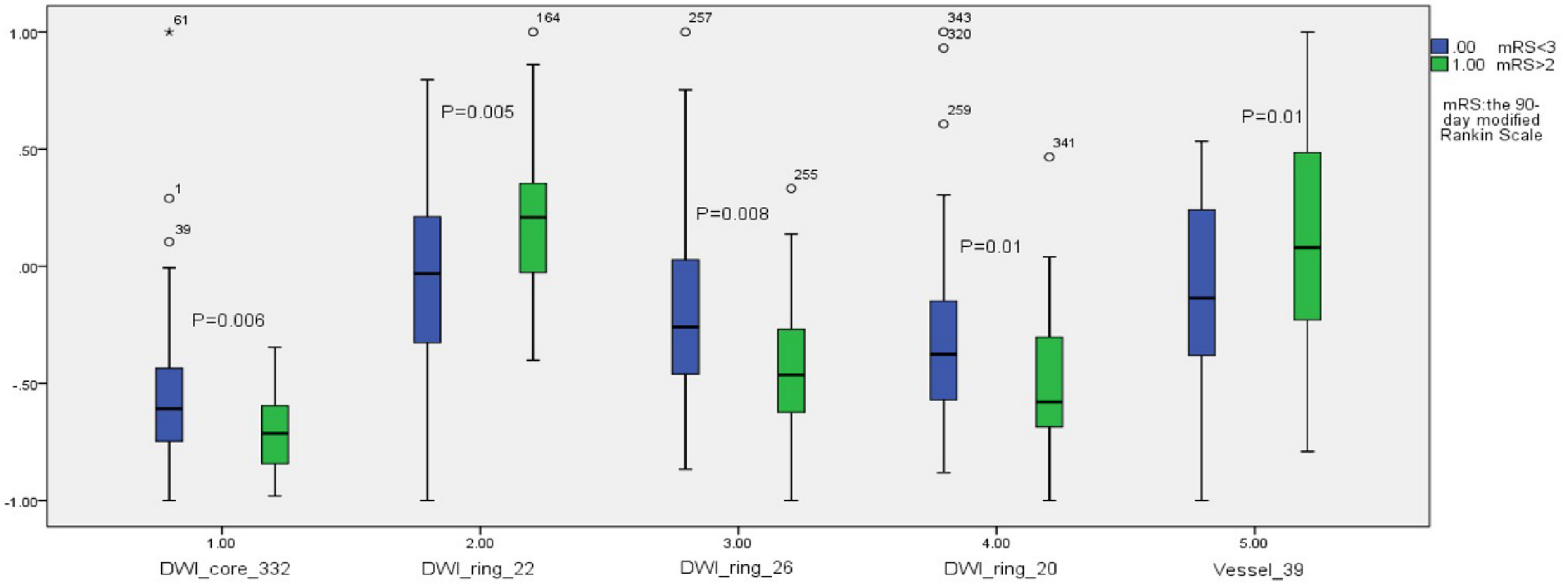
The statistical box plots of the five features with *P* ≤ 0.01.

Among the extracted features, 41 features showed statistical differences between the good and bad prognosis groups (*P* < 0.05). Figure 4 shows the statistical box plots of the five features with *P* <= 0.01. These five features included one ischemic lesion region radiomics feature (wavelet5.textures.gray-level size zone matrix.small zone low gray-level emphasis, WT5TGLSZMSZLGE), one vascular morphology feature (clustering coefficient), and three penumbra radiomics features (image.textures.gray-level co-occurrence matrix.homogeneity, ITGLCMH; image.textures.gray-level co-occurrence matrix.dissimilarity, ITGLCMD; image.textures.gray-level co-occurrence matrix.contrast, ITGLCMCONT).

Table 2 summarizes the overall prediction performance of the models on both the training and test sets. Of the comparison methods, the “Vessel” model relied solely on vascular morphology, the “DWI” model used only imaging omics, and the “Vessel+DWI” model integrated both feature types. These models achieved prediction accuracies of 0.684, 0.789, and 0.842 on the cross-validation set, and 0.655, 0.793, and 0.828 on the independent test set, respectively. The similar performance on the cross-validation and independent test sets demonstrates the robustness of the proposed models.

**Table 2:** The performance of the classifier.

Datasets	Methods	ACC	SEN	SPE	PPV	NPV
Cross validation	Vessel	0.684	0.500	0.784	0.556	0.744
	DWI	0.789	0.700	0.838	0.700	0.838
	Vessel+DWI	0.842	0.750	0.892	0.789	0.868
Independent test	Vessel	0.655	0.632	0.700	0.800	0.500
	DWI	0.793	0.684	1.000	1.000	0.625
	Vessel+DWI	0.828	0.789	0.900	0.937	0.692

**Figure 5:**
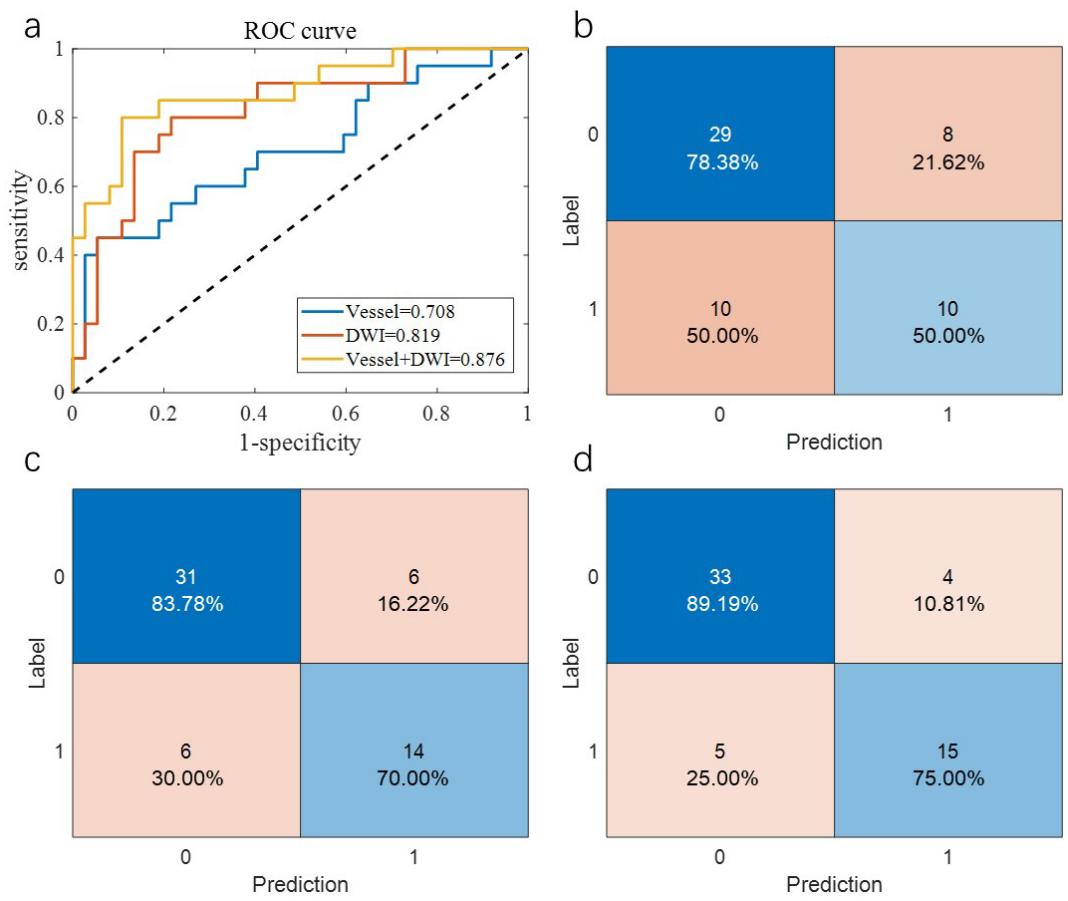
The AUC curves and confusion matrices of the three groups of models on the cross-validation set.

**Figure 6:**
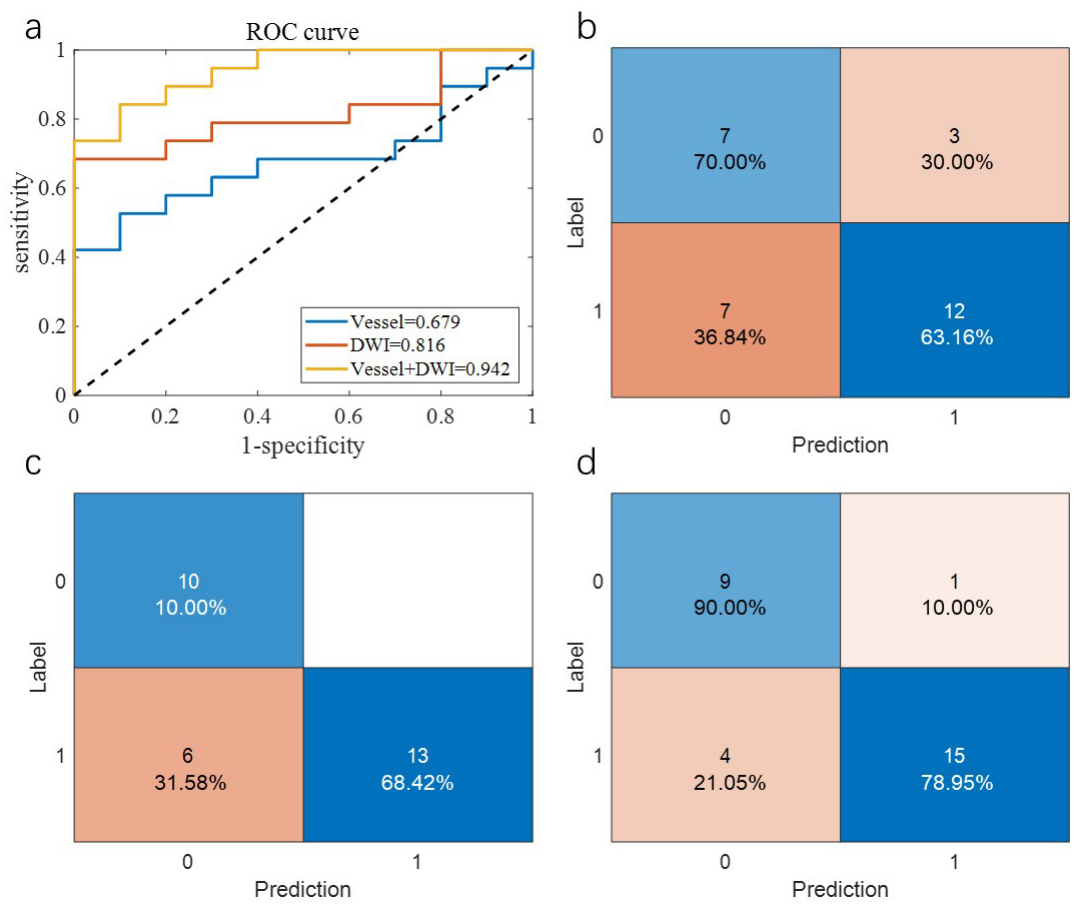
The AUC curves and confusion matrices of the three groups of models on the independent test set.

Additionally, the sensitivity and specificity differences for the “Vessel+DWI” model between the cross-validation and independent test sets were within 0.15, indicating a relatively balanced classification of positive and negative samples. Figures 5 and 6 illustrate the area under the curve (AUC) and confusion matrices for the three models on the cross-validation and independent test sets, respectively. Notably, the combined “Vessel+DWI” model achieved AUC values of 0.876 and 0.942 on the cross-validation and independent test sets, highlighting its superior predictive capability.

**Figure 7:**
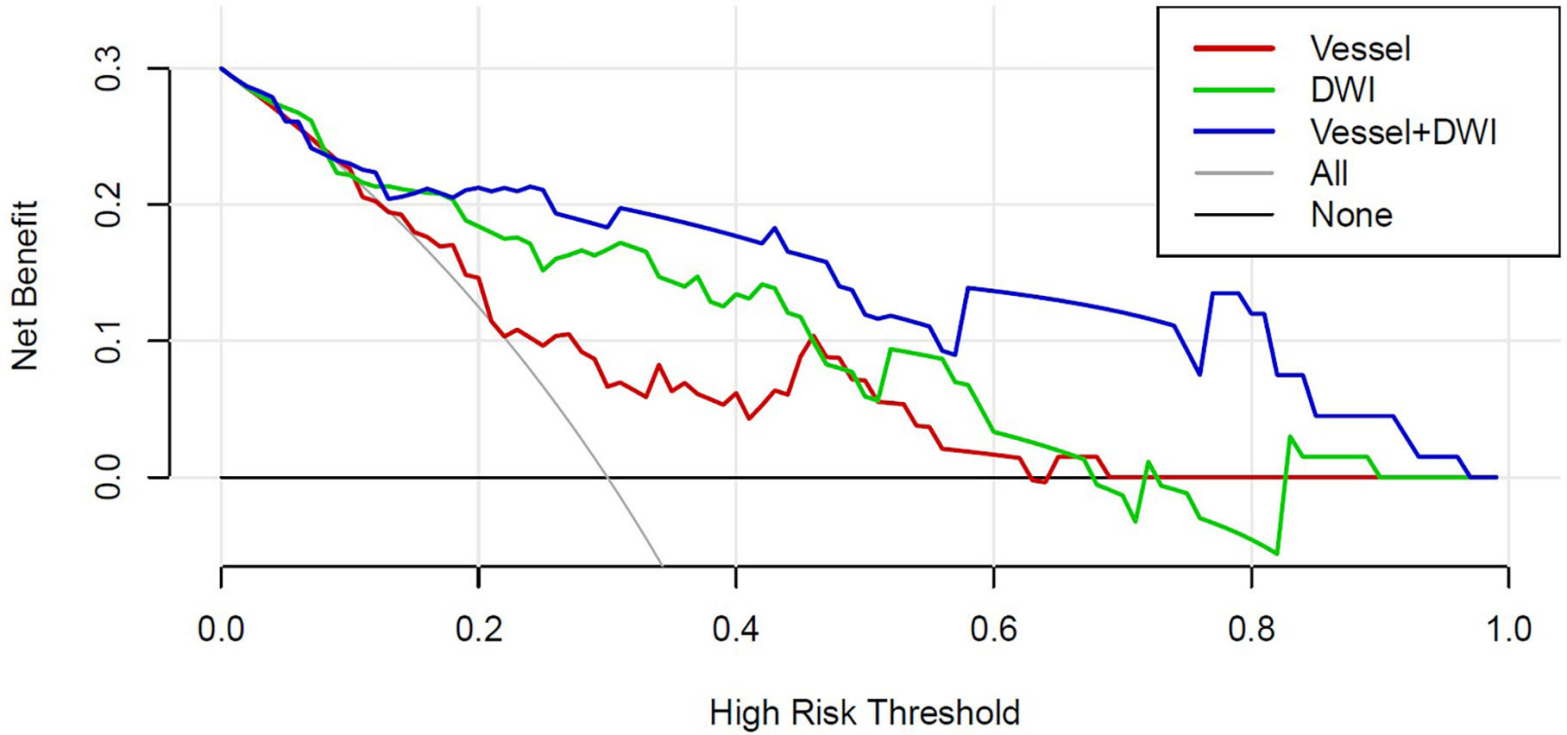
The decision curves of the three models on the independent test set.

**Figure 8:**
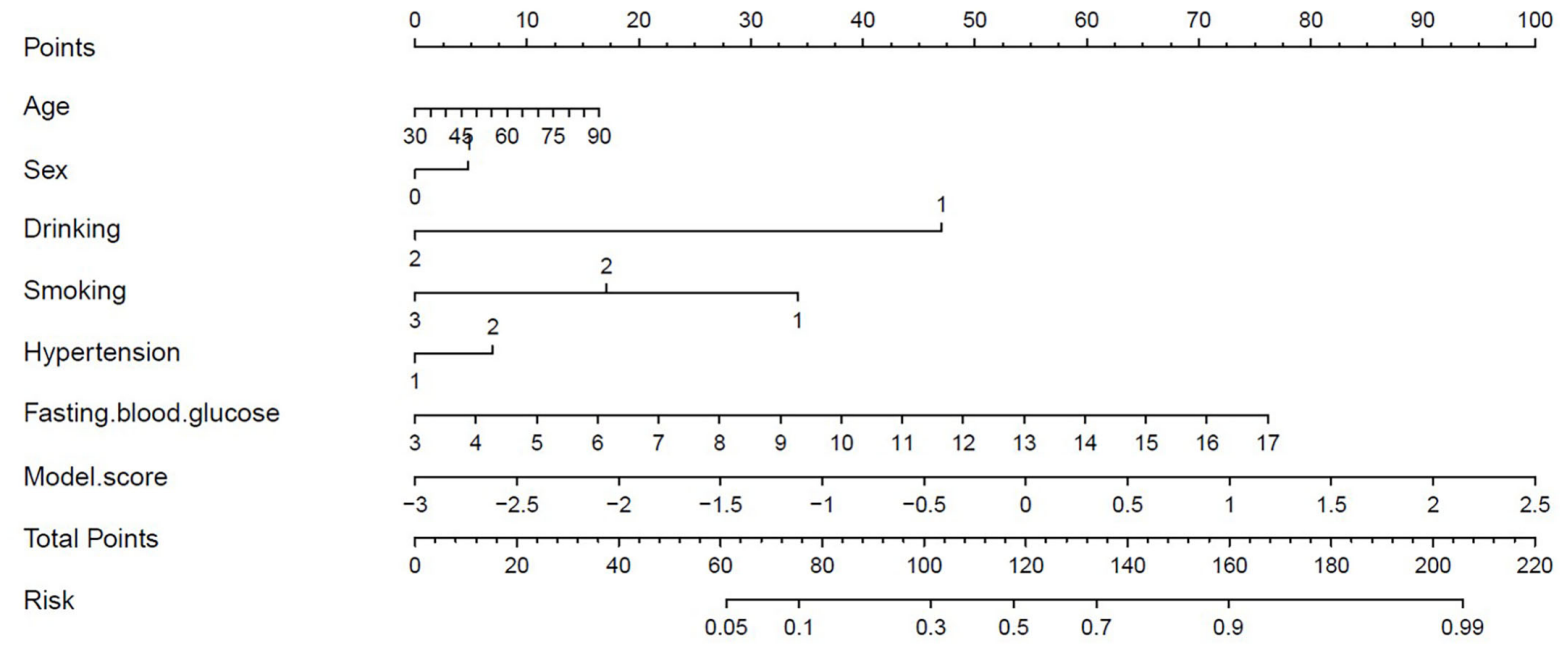
Diagnostic nomogram combining the combined model with clinical characteristics.

Figure 7 presents the decision curves for the three models on the independent test set, demonstrating that the combined “Vessel+DWI” model delivered higher net benefits compared to the other models. To further emphasize the model’s clinical applicability, a diagnostic nomogram was constructed by integrating the prediction scores of the “Vessel+DWI” model with key clinical indicators, as shown in Fig. 8. The calculated prediction probabilities from the nomogram offer valuable guidance for the clinical diagnosis and treatment of stroke.

## Discussion

Predicting 90-day mRS scores for stroke patients holds significant value for guiding treatment strategies. While numerous methods exist for predicting stroke prognosis based on brain CT or MRI images (Tanriverdi et al., 2016; Cao et al., 2025), challenges remain in the comprehensive quantification of whole-brain features. For instance, cerebrovascular-based approaches typically focus on the morphology of a few specific blood vessels but neglect the holistic evaluation of the entire cerebral vasculature (Brugnara et al., 2020). Furthermore, many evaluation methods rely on manual assessments by clinicians, which compromises the robustness and reproducibility of predictive models.

On the other hand, numerous studies have utilized radiomic features from the ischemic lesion region in DWI to predict stroke prognosis, but they often overlooked the impact of features derived from the penumbra region surrounding the ischemic lesion region. This limitation restricts the predictive performance of such models. To address these gaps, this study integrated morphological features of the whole-brain vasculature, radiomic features of the ischemic lesion region, and radiomic features of the penumbra region to predict 90-day mRS scores for stroke patients.

Previous research suggests that structural changes in the cerebrovascular system can distinguish healthy brains from diseased ones (Liu et al., 2018). Arterial anatomical features, such as curvature and bifurcation, influence hemodynamics and contribute to plaque formation and progression, which may trigger ischemic events (Leng, Wong & Liebeskind, 2014). Thus, changes in vascular structure are thought to reflect potential functional and pathophysiological alterations in the brain (Hu et al., 2023). In this study, the selected vascular morphological features effectively highlighted differences in the number of blood vessels between the infarct and healthy sides of the brain. Additionally, the findings suggest that minor reductions in small blood vessels may reliably predict infarction trends, providing valuable insights for clinical intervention.

In this study, a total of 1,066 imaging features were extracted. While high-throughput features can provide a comprehensive characterization of lesion conditions, such a large number of variables may increase computational burden and the risk of model overfitting. To address these challenges, we applied a sparse representation method for feature selection, followed by the construction of a non-parametric sparse representation classifier. Through this process, only 26 features were ultimately retained, which demonstrated the greatest discriminative power and yielded stable cross-validation performance. These results suggest that a compact and informative set of imaging biomarkers is sufficient to achieve accurate prognosis prediction while minimizing overfitting risk.

The experimental results demonstrated that all three feature types extracted in this study were statistically significant. Notably, the penumbra region exhibits more features with statistically significant differences (as shown in Fig. 4). The combined “Vessel + DWI” model achieved a prediction accuracy exceeding 0.82 on both the cross-validation and independent test sets, underscoring the effectiveness of the proposed approach. Furthermore, the combined models outperformed single-feature models, reinforcing the importance of the multi-modal feature integration proposed in this study.

**Figure 9:**
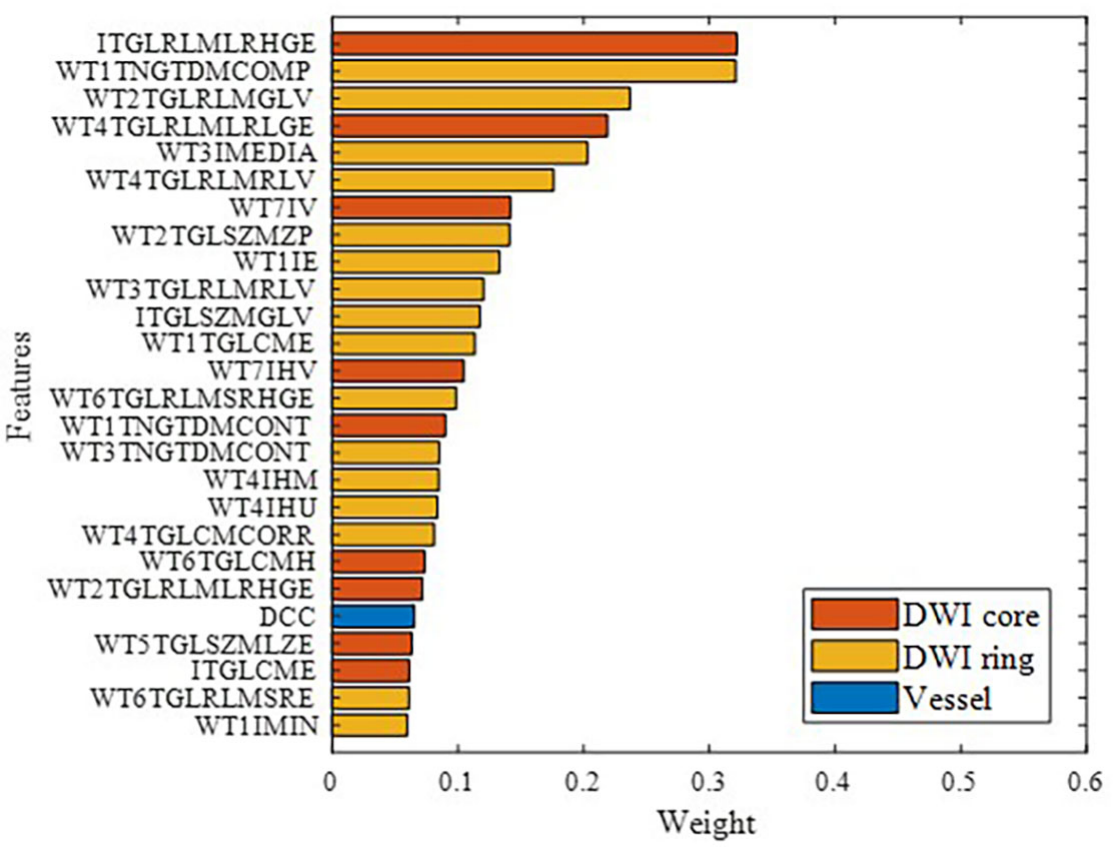
Modeling feature categories and importance ranking.

Figure 9 illustrates the features ultimately used for model building, ranked by importance, with different colors representing various feature categories. Overall, brain parenchymal features from DWI images were found to play a more critical role than cerebrovascular features. This may be because the characteristics of the ischemic lesion region and peri-ischemic region directly determine stroke prognosis.

This study has several limitations. First, we did not perform detailed morphological analyses of individual major vessels such as the MCA or ICA, which could provide additional prognostic insights. Second, delineation of the penumbra was approximated by outward expansion of the ischemic lesion on DWI rather than derived from perfusion imaging, due to the absence of consistent CTP data. Although sufficient for radiomics feature extraction, this approximation cannot fully replace perfusion-based characterization. Moreover, our current framework did not incorporate the TICI score. This was because our study cohort included both intravenous thrombolysis (IVT) and endovascular thrombectomy (EVT) patients. As the TICI grading system is applicable only to patients who undergo mechanical thrombectomy with angiographic evaluation, this parameter was unavailable for the entire population. While many previous studies on mRS prediction have focused exclusively on EVT cohorts (*e.g.*, Brugnara et al., 2020; Mistry et al., 2021), several recent radiomics and machine learning studies (*e.g.*, Li et al., 2025; Wei et al., 2024; Weng et al., 2023) have adopted a mixed-patient design that includes both IVT and EVT cases. Such a design enhances the generalizability and real-world applicability of prognostic models, as it reflects the true heterogeneity of acute ischemic stroke (AIS) patients encountered in clinical settings. In line with these studies, our aim was to develop a broadly applicable imaging-based prediction framework rather than a treatment-specific prognostic model. Therefore, the TICI grade was analyzed descriptively but was not incorporated as a predictive feature in the model. We acknowledge that future large-scale multicenter studies with homogeneous EVT data will enable the systematic integration of detailed reperfusion metrics—such as TICI grade and occlusion site—into model development, which is expected to further improve predictive accuracy, robustness, and interpretability.

## Conclusion

This study proposes a machine learning framework that integrates whole-brain vascular morphological features and radiomics features from both the ischemic lesion region and peri-ischemic region to predict the 90-day mRS scores for AIS patients. By extracting 1,066 features and employing sparse representation for feature selection, the final model achieved an accuracy of 82.8% and AUC of 0.942. The results highlight the importance of both vascular morphology and DWI-based radiomics features, especially from the penumbra region, in predicting stroke outcomes. This multimodal feature integration outperformed single-feature models, offering promising support for personalized treatment and early intervention, with strong potential for clinical application.

##  Supplemental Information

10.7717/peerj.20588/supp-1 Supplemental Information 1 Code for experimental results display Run ”results_show.m” to get the result data and figures in the paper.

10.7717/peerj.20588/supp-2 Supplemental Information 2 Extracted original feature data and model prediction result data Vascular morphological features and radiomics features of 86 cases.Multiple sets of comparison model prediction result data, including true labels, predicted labels and predicted scores

10.7717/peerj.20588/supp-3 Supplemental Information 3 STROBE checklist

## Additional Information and Declarations

### Data Availability

The following information was supplied regarding data availability: The code is available in the Supplemental Files.

## References

[ref-1] Brugnara G, Neuberger U, Mahmutoglu MA, Foltyn M, Herweh C, Nagel S, Schönenberger S, Heiland S, Ulfert C, Ringleb PA, Bendszus M, Möhlenbruch MA, Pfaff JAR, Vollmuth P (2020). Multimodal predictive modeling of endovascular treatment outcome for acute ischemic stroke using machine-learning. Stroke.

[ref-2] Cao R, Lu Y, Li W, Yu F, Hu S, Chen K, Wang G, Sun C, Ma Q, Zhang M, Chen J, Lu J (2025). Dynamic CTA-based whole-brain arterial-venous collateral assessment for predicting futile recanalization in acute ischemic stroke. Aging and Disease.

[ref-3] Chen W, Qin Y, Yang S, Yang L, Hou Y, Hu W (2023). Effect of leukoaraiosis on collateral circulation in acute ischemic stroke treated with endovascular therapy: a meta-analysis. BMC Neurology.

[ref-4] Fu F, Wei J, Zhang M, Yu F, Xiao Y, Rong D, Shan Y, Li Y, Zhao C, Liao F, Yang Z, Li Y, Chen Y, Wang X, Lu J (2020). Rapid vessel segmentation and reconstruction of head and neck angiograms using 3D convolutional neural network. Nature Communications.

[ref-5] Hu P, Zhou H, Yan T, Miu H, Xiao F, Zhu X, Shu L, Yang S, Jin R, Dou W, Ren B, Zhu L, Liu W, Zhang Y, Zeng K, Ye M, Lv S, Wu M, Deng G, Hu R, Zhan R, Chen Q, Zhang D, Zhu X (2023). Deep learning-assisted identification and quantification of aneurysmal subarachnoid hemorrhage in non-contrast CT scans: development and external validation of hybrid 2D/3D UNet. NeuroImage.

[ref-6] Huo X, Ma G, Tong X, Zhang X, Pan Y, Nguyen TN, Yuan G, Han H, Chen W, Wei M, Zhang J, Zhou Z, Yao X, Wang G, Song W, Cai X, Nan G, Li D, Wang AY-C, Ling W, Cai C, Wen C, Wang E, Zhang L, Jiang C, Liu Y, Liao G, Chen X, Li T, Liu S, Li J, Gao F, Ma N, Mo D, Song L, Sun X, Li X, Deng Y, Luo G, Lv M, He H, Liu A, Zhang J, Mu S, Liu L, Jing J, Nie X, Ding Z, Du W, Zhao X, Yang P, Liu L, Wang Y, Liebeskind DS, Pereira VM, Ren Z, Wang Y, Miao Z (2023). Trial of endovascular therapy for acute ischemic stroke with large infarct. New England Journal of Medicine.

[ref-7] Lei H, Wu X, Ambler G, Werring D, Fang S, Lin H, Huang H, Liu N, Du H (2024). Association between perivascular spaces burden and future stroke risk in ischemic stroke and transient ischemic attack: a systematic review and meta-analysis. European Neurology.

[ref-8] Leng X, Wong KS, Liebeskind DS (2014). Evaluating intracranial atherosclerosis rather than intracranial stenosis. Stroke.

[ref-9] Li G, Zhang Y, Tang J, Chen S, Liu Q, Zhang J, Shi S (2025). Diffusion-weighted imaging—based radiomics features and machine learning method to predict the 90-day prognosis in patients with acute ischemic stroke. The Neurologist.

[ref-10] Liu Y, Ghassemi P, Depkon A, Iacono MI, Lin J, Mendoza G, Wang J, Tang Q, Chen Y, Pfefer TJ (2018). Biomimetic 3D-printed neurovascular phantoms for near-infrared fluorescence imaging. Biomedical Optics Express.

[ref-11] Lv Z, Huang H, Sun W, Lei T, Benediktsson JA, Li J (2023). Novel enhanced UNet for change detection using multimodal remote sensing image. IEEE Geoscience and Remote Sensing Letters.

[ref-12] Mistry EA, Yeatts S, De Havenon A, Mehta T, Arora N, De Los Rios La Rosa F, Starosciak AK, Siegler JE, Mistry AM, Yaghi S, Khatri P (2021). Predicting 90-day outcome after thrombectomy: baseline-adjusted 24-hour NIHSS is more powerful than NIHSS score change. Stroke.

[ref-13] Ronneberger O, Fischer P, Brox T (2015). U-Net: convolutional networks for biomedical image segmentation.

[ref-14] Seker F, Pereira-Zimmermann B, Pfaff J, Purrucker J, Gumbinger C, Schönenberger S, Bendszus M, Möhlenbruch MA (2020). Collateral scores in acute ischemic stroke: a retrospective study assessing the suitability of collateral scores as standalone predictors of clinical outcome. Clinical Neuroradiology.

[ref-15] Tang T, Jiao Y, Cui Y, Zhao D, Zhang Y, Wang Z, Meng X, Yin X-D, Yang Y-J, Teng G, Ju S (2020). Penumbra-based radiomics signature as prognostic biomarkers for thrombolysis of acute ischemic stroke patients: a multicenter cohort study. Journal of Neurology.

[ref-16] Tanriverdi Z, Gocmen R, Oguz KK, Topcuoglu MA, Arsava EM (2016). Elevations in tissue fluid-attenuated inversion recovery signal are related to good functional outcome after thrombolytic treatment. Journal of Stroke and Cerebrovascular Diseases.

[ref-17] Vagal A, Aviv R, Sucharew H, Reddy M, Hou Q, Michel P, Jovin T, Tomsick T, Wintermark M, Khatri P (2018). Collateral clock is more important than time clock for tissue fate: a natural history study of acute ischemic strokes. Stroke.

[ref-18] Vallières M, Freeman CR, Skamene SR, El Naqa I (2015). A radiomics model from joint FDG-PET and MRI texture features for the prediction of lung metastases in soft-tissue sarcomas of the extremities. Physics in Medicine and Biology.

[ref-19] Van Der Hoeven EJ, McVerry F, Vos JA, Algra A, Puetz V, Kappelle LJ, Schonewille WJ, on behalf of the BASICS registry investigators (2016). Collateral flow predicts outcome after basilar artery occlusion: the posterior circulation collateral score. International Journal of Stroke.

[ref-20] Wang Z, Li J, Wang C, Yao X, Zhao X, Wang Y, Li H, Liu G, Wang A, Wang Y (2013). Gender differences in 1-year clinical characteristics and outcomes after stroke: results from the China National Stroke Registry. PLOS ONE.

[ref-21] Wang H, Sun Y, Zhu J, Zhuang Y, Song B (2022). Diffusion-weighted imaging-based radiomics for predicting 1-year ischemic stroke recurrence. Frontiers in Neurology.

[ref-22] Wei L, Pan X, Deng W, Chen L, Xi Q, Liu M, Xu H, Liu J, Wang P (2024). Predicting long-term outcomes for acute ischemic stroke using multi-model MRI radiomics and clinical variables. Frontiers in Medicine.

[ref-23] Weng S, Sun X, Wang H, Song B, Zhu J (2023). A new method for predicting the prognosis of ischemic stroke based vascular structure features and lesion location features. Clinical Imaging.

[ref-24] Wu G, Chen Y, Wang Y, Yu J, Lv X, Ju X, Shi Z, Chen L, Chen Z (2018). Sparse representation-based radiomics for the diagnosis of brain tumors. IEEE Transactions on Medical Imaging.

[ref-25] Wu G, Lin J, Chen X, Li Z, Wang Y, Zhao J, Yu J (2019a). Early identification of ischemic stroke in noncontrast computed tomography. Biomedical Signal Processing and Control.

[ref-26] Wu G, Shi Z, Chen Y, Wang Y, Yu J, Lv X, Chen L, Ju X, Chen Z (2019b). A sparse representation-based radiomics for outcome prediction of higher grade gliomas. Medical Physics.

[ref-27] Xu J, Wang A, Meng X, Yalkun G, Xu A, Gao Z, Chen H, Ji Y, Xu J, Geng D, Zhu R, Liu B, Dong A, Mu H, Lu Z, Li S, Zheng H, Chen X, Wang Y, Zhao X, Wang Y (2021). Edaravone dexborneol versus edaravone alone for the treatment of acute ischemic stroke: a phase III, randomized, double-blind, comparative trial. Stroke.

[ref-28] Zhang X, Tang Y, Xie Y, Ding C, Xiao J, Jiang X, Shan H, Lin Y, Li C, Hu D, Li T, Sheng L (2017). Total magnetic resonance imaging burden of cerebral small-vessel disease is associated with post-stroke depression in patients with acute lacunar stroke. European Journal of Neurology.

